# Utilization of and Barriers to HIV and MCH Services among Community ART Group Members and Their Families in Tete, Mozambique

**DOI:** 10.1155/2013/937456

**Published:** 2013-07-17

**Authors:** Diederike Geelhoed, Tom Decroo, Sergio Dezembro, Humberto Matias, Faustino Lessitala, Fausto Muzila, Luisa Brumana, Emanuele Capobianco

**Affiliations:** ^1^International Centre for Reproductive Health-Mozambique, Rua José Macamo 269–1A, Maputo, Mozambique; ^2^Médecins Sans Frontières, Avenida Eduardo Mondlane 38, Tete, Mozambique; ^3^Changara District Health Services, Changara Sede, Tete, Mozambique; ^4^UNICEF, 1440 Avenida do Zimbabwe, Maputo, Mozambique

## Abstract

Mozambique continues to face many challenges in HIV and maternal and child health care (MCH). Community-based antiretroviral treatment groups (CAG) enhance retention to care among members, but whether such benefits extend to their families and to MCH remains unclear. In 2011 we studied utilization of HIV and MCH services among CAG members and their family aggregates in Changara, Mozambique, through a mixed-method assessment. We systematically revised all patient-held health cards from CAG members and their non-CAG family aggregate members and conducted semistructured group discussions on MCH topics. Quantitative data were analysed in EPI-Info. Qualitative data were manually thematically analysed. Information was retrieved from 1,624 persons, of which 420 were CAG members (26%). Good compliance with HIV treatment among CAG members was shared with non-CAG HIV-positive family members on treatment, but many family aggregate members remained without testing, and, when HIV positive, without HIV treatment. No positive effects from the CAG model were found for MCH service utilization. Barriers for utilization mentioned centred on insufficient knowledge, limited community-health facility collaboration, and structural health system limitations. CAG members were open to include MCH in their groups, offering the possibility to extend patient involvement to other health needs. We recommend that lessons learnt from HIV-based activism, patient involvement, and community participation are applied to broader SRH services, including MCH care.

## 1. Introduction

A decade after its large-scale introduction, countries in poor resourced sub-Saharan Africa (SSA), including Mozambique, continue to face challenges to scale-up antiretroviral therapy (ART). It is estimated that ART coverage is still only around 50%, while HIV-related mortality remains high, not only because of low-treatment coverage, but also due to poor retention of patients initiated on treatment [1–3]. Prevention of mother-to-child transmission (PMTCT) fares little better in SSA, as the uptake of PMTCT and early infant diagnosis continue to be unsatisfactory [1, 4]. As such, there is an urgent need for innovative strategies to offer ART to more people, including HIV-positive pregnant women, as well as to retain them in care [5]. Simultaneously, Mozambique and other SSA countries face difficulties in the field of maternal and child health (MCH) and are struggling to achieve their MDG 4 and 5 commitments [6–8]. The integration of HIV with other components of reproductive health, including MCH, is increasingly being recommended, aiming at a holistic approach towards sexual and reproductive health (SRH) through family-centred care [4]. Moreover, the HIV care model needs to adapt to lessons learnt from chronic disease care and attribute a more central role to patients [9–11].

In order to improve retention of patients receiving ART, Mozambique recently introduced innovative community-based groups for antiretroviral treatment (community ART groups, CAG). The methodology and results of this patient-driven care model have been described elsewhere and include good retention in care among CAG members (97.5% at 12-month follow-up) [12]. Self-forming groups of a maximum of six patients perform four key tasks: monthly distribution of antiretroviral medication among group members in the community, provision of adherence and social support, monitoring and reporting of treatment outcomes, and ensuring 6 monthly clinical consultations for each group member. The model uses social support networks to enhance social capital as a resource built on trust, cooperation, reciprocity, and sociability [13]. In particular for HIV care supportive peer relationships have the capacity to decrease stigma and improve adherence [14–16]. Moreover, when chronic disease care is provided by and integrated in the community, social outcomes may be improved [17]. It is, however, not yet clear whether these benefits for HIV care are extended to the families of CAG members and to other aspects of SRH, such as MCH. We therefore studied the utilization of HIV and MCH services and outcomes among family aggregates of CAG members in rural Mozambique.

## 2. Methods

A quantitative and qualitative assessment was implemented between April and June 2011 in the rural district of Changara, Tete Province, in Central Mozambique, where the CAG model has been introduced since 2008. A district-wide mobilization of members from all 105 CAG registered in the district at the time was realized through the established communication channels within the groups, inviting members to participate in one of 12 meetings in seven different localities throughout the district. During these gatherings, a systematic revision of a variety of patient-held health cards related to MCH from the CAG members and the non-CAG members of their family aggregates was carried out by one of the principal investigators (medical doctor). We distinguished two types of CAG members: formal members, who are HIV positive and receive antiretroviral treatment within a CAG, and social members, who are also HIV positive but who do not receive antiretroviral treatment and participate in the CAG for the social dynamics, while receiving individual follow-up for the prevention of opportunistic infections. For the purpose of this study, the family aggregate was defined as comprising all persons who habitually eat and sleep in the same household as the CAG member. Variables collected through questioning of CAG members and observation of their health cards included the following.

- For all participants: utilization of HIV counselling and testing and test results, follow-up, and treatment for HIV (if HIV positive).
- For women of reproductive age (15–49 years): utilization of health care in pregnancy and childbirth, modern contraceptive use, and tetanus immunization status.
- For children under the age of 5 years: attendance of under-five child care, including immunization and growth monitoring, as well as follow-up of HIV-exposed infants and children with low weight-for-age.

The quantitative data were digitalised, verified, and analysed in EPI-Info, version 3.5.1. 

In addition, a group discussion of two- to three-hour duration based on a semistructured questionnaire was conducted by one of two experienced facilitators, debating knowledge, and perceptions on a series of reproductive health care topics, with an emphasis on pregnancy and childbirth, contraception, and child health and nutrition. Both facilitators have longstanding experience interacting with the CAG and their members in Changara and received additional information and guidance on MCH matters from one of the principal investigators (medical doctor) before and during the period over which the group meetings were held. The discussions were conducted in the local language and transcribed into Portuguese by two scribes, who afterwards digitalised the information in Microsoft Office Word. The transcriptions were double-checked and, when necessary, corrected by the facilitators and principal investigators. These qualitative data were thematically organised in Microsoft Excel and manually analysed by the study team, based upon general consensus. On-going analysis was carried out during the period of data collection, resulting in punctual adaptations of the discussion guides in order to enrich the obtained information and understanding of all study topics, and data collection was continued till saturation was reached.

The study adhered to international ethical principles for health research. It was designed and implemented by the District Health Authorities within their routine health program management practices. CAG is a dynamic recommended by the Ministry of Health in Mozambique to increase patient participation to overcome barriers to adhesion to and retention in care. Formal ethical approval was not looked for as meetings, debates, and interactions between health care providers, facilitators, and CAG members form part of the normal activities of the CAG. Participation in CAG and in meetings among CAG members in the community is voluntary. The methods and purpose of our research were explained orally to participants in the group meetings for this study, and they gave their verbal consent before the start of every debate and the review of their health cards. No information regarding personal identification was collected. When significant health problems were identified, the CAG member concerned was informed and advised to seek care. In serious cases, clinical attendance was immediately arranged through the local health facility.

## 3. Results

### 3.1. Quantitative Results

In the group meetings participated 367 formal and social CAG members affiliated with 92 CAG (88% of all CAG in the district). These informants provided information on 330 family aggregates. The 330 family aggregates consisted of a total of 1,624 persons, including 420 CAG members. Of the 420 CAG members, 358 were formal members (76% of all persons registered as a member of a CAG in Changara) and 62 social members. Demographic details of these participants are presented in Table 1. 

#### 3.1.1. HIV Care Utilisation among CAG Members and Their Family Aggregates

Table 2 details our findings on utilization of HIV services. As expected, all CAG members were HIV positive and receiving either preventive care for opportunistic infections or ART, to which they showed excellent retention, all having taken their turn for clinical care and collection of medication for themselves and the members of their CAG within the previous 6 months. 

Among the non-CAG members of their family aggregates, 32% of men and 41% of women had received counselling and testing for HIV, with a positivity rate of 19% and 16%, respectively. Many of the non-CAG HIV-positive adult family members (men 79%; women 69%) reportedly did attend health care services to receive preventive care for opportunistic infections or ART, and they had the same good retention in care as the CAG members. The non-CAG children aged 6–14 years and under-fives had been tested in, respectively, 44% and 50%, in both groups with a positivity rate of 5%. All non-CAG HIV-positive children aged 6 to 14 years and 75% of HIV-positive under-fives were reportedly receiving treatment (preventive care for opportunistic infections or ART) with good adherence to care. Among infants from HIV-positive mothers, 79% of whom were registered in the follow-up programme for HIV-exposed infants, 67% had been tested, while none had been found to be HIV positive.

#### 3.1.2. MCH Service Utilisation among Women of Reproductive Age

Table 3 presents the study findings regarding care for pregnancy and childbirth, contraception, and tetanus immunization among women of reproductive age. Many women had experienced childbirth at least once, CAG members (89%) more often than non-CAG members (55%), probably related to their older age (mean age 33,4 years and 22,3 years, resp.). Their most recent childbirth occurred frequently with skilled attendance (78% and 83% of CAG and non-CAG members, resp.). At the time of data collection 7% of CAG members and 5% of non-CAG members reported to be pregnant (including early, nonconfirmed pregnancies); 61% of pregnant CAG members and 86% of pregnant non-CAG members had started antenatal care in their current pregnancy. Of the pregnant CAG members 89% were receiving antiretroviral therapy, started already before their current pregnancy, protecting them also against HIV transmission to their child, but neither of the two HIV-positive pregnant non-CAG members were receiving any medication for the prevention of mother-to-child transmission. Use of modern contraceptive methods was limited in all women of reproductive age (16% and 19% of CAG and non-CAG members, resp.), usually injectable or oral contraceptives. Condom use as a contraceptive method was reported just three times (one CAG member and two non-CAG members). Less than half of the participants brought documentation regarding tetanus immunization in women of reproductive age, which was up-to-date in 41% of CAG members and 38% of non-CAG members. 

#### 3.1.3. MCH Service Utilisation among Children under Five Years of Age

Results regarding immunization, growth monitoring, and nutrition in the under-fives included in our study population are presented in Table 4. Only four under-fives were reportedly social CAG members, and as such these results are not disaggregated for CAG and non-CAG members. Of the children under the age of one year 72% had received all immunizations recommended for their age according to the national vaccination calendar. Children aged 12–59 months were fully immunized in 95%. Babies under 6 months of age were exclusively breastfed in 70%. Growth monitoring had been performed within the previous month in 79% of children under the age of one year, and within the previous 3 months in 52% of children aged 12–59 months. A low weight-for-age at the most recent visit had been documented on the health cards of 15% of children under the age of one year and of 49% of children aged 12–59 months. The health cards of these children with low weight-for-age indicated in 75% and 23%, respectively, referral to the at-risk child clinic for further follow-up. Those same health cards showed that Vitamin A supplementation and deworming were up-to-date according to the national guidelines at the time of the study in, respectively, 62% and 61% of children of eligible age. 

### 3.2. Qualitative Results

The 367 CAG members participating in the quantitative assessment also participated in the qualitative data collection. During the discussions in the group meetings, the three main MCH areas were addressed: pregnancy and childbirth; contraception and tetanus immunization for women of reproductive age; and health and nutrition for young children. Three main themes emerged: (1) knowledge and perceptions in the community; (2) interface between community and health services; and (3) performance of the health services.

#### 3.2.1. Knowledge and Perceptions in the Community

Many participants appeared well informed regarding care during pregnancy and childbirth as recommended by the health services, and they recognized the importance of those health services for pregnant women and women in childbirth. Nonetheless, they also mentioned that some others in the community were less convinced of the benefit of such health services and that therefore not everybody showed interest in using the health services for care during pregnancy and childbirth.

*“Nowadays there are many diseases, and attending in the hospital is good, because there they provide a health check and care to guarantee a healthy development of the baby and a safe childbirth. In case of disease, when we comply with the antenatal care visits, it is unlikely that this disease transmits from the mother to the baby. [Attending the hospital] is also important because sometimes a blood transfusion is required during or after childbirth, and this prevents death.” Participant in Marara-Centro, 25th May, 2011. *

Modern contraceptive methods formed another topic on which participants were quite knowledgeable, reciting extensively on the range of available methods and their advantages. At the same time, many expressed serious doubts and concerns regarding the use of contraceptives, related to side effects (such as vaginal blood loss) or fears that such use might adversely affect their fertility in the future. “*Contraceptives damage the uterus*” was a frequently heard remark. In addition, contraceptive use arose as a potential source of discord between couples, as the participants claimed that husbands do not always agree with their wives' wishes for contraception, fearing a loss of control over their sexual activities. It was suggested that many women have to practice family planning in secret. Therefore, the participants were not very confident that contraception would actually be a good option for them.

*[Why is it that some women do not practice family planning?] “Because they are afraid that they will not be able to conceive again, and others because the husbands forbid it, they allege that if a woman uses family planning, she will prostitute herself with other men, because she knows she will not be able to get pregnant.” Participant in Luenha, 15th June, 2011. *

Much less knowledge was displayed regarding tetanus immunization for women of reproductive health. Though the participants knew about tetanus immunization in pregnant women, they were not clear about its purpose, and apparently neither had they any idea about the recommended immunization calendar for tetanus in women of reproductive age.

The need for extra care for young children, such as immunization and growth monitoring, was well known and accepted by the participants in the group meetings. However, it appeared that the participants considered this particular care for young children only necessary till they were quite grown already, that is, until the age of two years or so, while the health services recommend such care from birth till the age of five years. Moreover, a young child for our participants appeared to be only the youngest child: when a mother has several under-fives, she often would take only the youngest to the health facility for growth monitoring and check-up, and any older children would be taken to attend the health services only in case of illness. Despite this, the participants generally showed great interest and felt the need to learn more about care for young children, in particular about ways to improve their children's care which would be feasible in their environment and within their means, and especially in the field of child nutrition.

*[Why do people no longer bring their children for weighing when they reach the age of two or three years?] “Many people do no longer take them for weighing because they see that the child is already quite big, and is looking good, and then they think there is no need anymore to take them for weighing when the child is not sick …” Participant in Changara-Sede, 17th June, 2011.*

#### 3.2.2. Interface between Community and Health Services

The long travelling distances to and from health facilities and the lack of means for transportation, especially in emergencies, were highlighted on several occasions as major barriers for the use of health services, in particular during pregnancy and childbirth. Moreover, many participants considered that the great efforts required to reach a health facility for care were not always sufficiently compensated by the quality of care received there, in pregnancy and childbirth, but also in childcare.

*“A lack of transport, here it is not possible to hire a car, and [women in childbirth] therefor stay a long time at home, where they end up giving birth. The few cars that sometimes are available charge a lot of money, which we do not have. In the health centre there is no place for pregnant women to wait for their childbirth, and the nurses sometimes are not present in the health centre, so that does not compensate the efforts we make in traveling from home to the health facility, when upon arrival there we do not encounter the nurse.” Participant in Dzunga, 6th June, 2011.*

The participants thought it might be useful to try to bring health care closer to the people requiring it. It was suggested to look at the option of widening the range of activities in the CAG for this purpose and to engage CAG members in growth monitoring and prophylaxis for opportunistic infections in HIV-exposed infants and HIV-positive family members. In the case of contraception, though they considered that contraceptive use might be a delicate secret, an analogue was found with the secret of being HIV positive, which was overcome within the social group dynamics:

*[What support could the group provide for those people who need family planning?] “We could organize the groups so that one person could collect the family planning for the others, as we do with the antiretroviral medication. Family planning is often a secret in the family, similar as HIV/AIDS. In the beginning it was also a secret to take antiretroviral drugs. Nowadays we are organized in groups, and we manage to help ourselves, so we should proceed in a similar manner for family planning.” Participant in Cachembe, 20th May, 2011.*

#### 3.2.3. Performance of the Health Services 

During all group discussions it became apparent that the CAG members experience and are disappointed by the lack of qualified human resources, of commodities including drugs, and of equipment and means of transport such as ambulances, prevailing in their health services. Complaints about the unavailability of prescribed medications and other commodities, such as HIV tests, insecticide-impregnated nets, and products for nutritional supplementation and treatment, appeared during practically all debates, especially when referring to the more peripheral health facilities.

* “Sometimes in the health centre there is a stock out of medications, family planning, or the nurse is absent. There should be immunizations always, HIV tests, because here they do not test anymore for HIV when mothers go to start antenatal care, and the number of nurses is not sufficient.” Participant in Missawa, 8th June, 2011. *

In addition, the difficulties encountered during visits to health facilities, where large crowds and long queues are common, contribute to the feeling that such visits are not always worthwhile. The participants also remarked repeatedly that they consider that some health care providers, particularly certain staff in the MCH department and pharmacy at the district hospital, do not attend them with adequate manners and respect, which leads them to avoid seeking care.

* [What are barriers to antenatal care visits?] “Many do not go to the hospital, because the midwives beat them, and get angry with them, and that way they intimidate the people who attend the hospital.” [All participants concurred with this remark.] Participant in Changara-Sede, 13th June, 2011.*

*“Many mothers are afraid to talk, because it appears they are just complaining, but it is true that this is happening, that bad attendance occurs in the hospital. Not everywhere, but it does occur in the MCH sector and in the Pharmacy. Many people turn around when they are on the way, when they hear that a certain person is on duty there.” Participant in Changara-Sede, 17th June, 2011.*

## 4. Discussion

Our study confirms that the CAG model as implemented in Changara, one of the districts which pioneered this patient-centred care model, is associated with very good compliance to HIV care and treatment among the group members, as has been reported before [12]. The study findings also suggest that this habit of good compliance to HIV care and treatment is shared with HIV-positive persons receiving individual treatment among the family aggregates of the CAG members, as all known HIV-positive persons on treatment in our study population reportedly complied with the required regular clinical consultations and medication collection. However, many non-CAG family members remained without counselling and testing, and a considerable number of HIV-positive family members remained without HIV treatment and care. Men had the lowest utilization of counselling and testing (32%), while those who were tested resulted often HIV-positive (19%) and eligible for ART (71%). This probably reflects the known reluctance of men in Mozambique and similar areas in accessing health services unless they are really ill, as reported by other studies [18, 19]. Yet even women, who use health services more regularly for themselves or their children, were only counselled and tested in 41%, with a high positivity rate of 16%. A considerable proportion of these non-CAG HIV-positive adult family members did not access HIV treatment, despite their respective CAG members' knowledge of the positive HIV test. It appears that the positive effects of the CAG support are centred in the compliance to ART care, rather than also addressing utilization of testing services and initiation of HIV treatment. It might be possible to strengthen the CAG dynamics towards the inclusion of counselling and testing through community-based VCT services, as well as towards increased utilization of HIV care and treatment for those tested HIV positive through improved interpersonal communication and support among the adults within family aggregates and the wider community. 

In our study population, utilization of counselling and testing increased with younger age of the family members, and in parallel their HIV-positivity rate reduced. Despite this, a considerable number of children, even when known HIV exposed, had not been tested. In view of the need to accelerate access to paediatric HIV treatment, which in Mozambique till date has a much lower coverage than adult HIV treatment [2], it would be important to actively promote among the CAG members the use of early infant diagnosis and HIV testing for their children at all possible entry points in health facilities and to ensure universal access to treatment for those children testing HIV positive. Regarding the prevention of paediatric HIV infection, it appears that the CAG model does make a positive contribution, as 89% of pregnant CAG members received medication effective for PMTCT, although this might rather be an unintentional benefit from the large proportion of women of reproductive age on ART within the CAG. As such, encouraging counselling, testing, and access to HIV treatment for all women of reproductive age might expand this PMTCT effect to the family aggregates and the wider community. 

Our data regarding utilization and outcomes of MCH services among the CAG members and their family aggregates appear to largely resemble those for the general population as reported routinely by the district and provincial health system. Only the proportions of skilled attendance at birth and of exclusive breastfeeding of babies under 6 months of age seem relatively high in our study population. However, these might relate to certain general characteristics of the study population, such as the very high HIV prevalence and a general motivation to use formal health services, rather than to particular positive effects from the CAG model. However, the CAG members in our study made it clear that they were interested in and open to an expansion of the health topics dealt with within their groups. This patient-driven care model offers the possibility to extend the activated level of patient involvement to other needs than HIV care and treatment among people living with HIV/AIDS. Lessons learnt from HIV-based activism, patient involvement, and community participation might be applied to broader SRH services, including MCH care. Community and patient participation increases the sharing of knowledge, treatment experiences, and acquired self-management skills among peers and their family aggregates in the community [5, 10]. Moreover, the social interactions may induce the necessary motivation to adhere to health services and to initiate care. More informed and more motivated people are more likely to acquire health promoting skills and practice health seeking behaviour [20]. There is a need for honest and open information at an appropriately simplified but not unsophisticated level within the CAG about wider SRH, including MCH topics. For example, in the case of family planning, the need exists for an honest and open dialogue on its pros and cons, including ways to safeguard fertility for the future and what to do in case of infertility.

Our participants related the lack of MCH service utilization to cultural perspectives leading to lack of trust, to a lack of appropriate information in the community, and to structural barriers such as a lack of transport, unavailability of MCH personnel, stock-outs of drugs and equipment, and the fragmented delivery of MCH services. For obvious ethical reasons such structural health system limitations need to be addressed in order to respond appropriately to any created demand for services. General health system strengthening is essential in Mozambique and many other countries in SSA, for health systems to be able to offer services of a quality which is worthy of people's trust and which generates confidence in its use among the population. 

Despite the wide participation in our study among the CAG members in Changara, we did not manage to collect information on all family aggregate members, as not all health cards were available for review. Hence, the denominators of many of the variables presented in the tables are smaller than the total number of CAG and aggregate members, sometimes leading to very small numbers in certain subgroups. It is possible that this has caused a distortion of our results, probably in a positive direction, as CAG members with knowledge and cards on the health status of their family aggregate members likely represented families with more motivation for health care utilization than those without. In addition, we do not present a statistical comparison between the various groups in our study population, as its interpretation would be difficult due to the obvious interdependence between the CAG members and their non-CAG family aggregates. 

As such, our quantitative results should be interpreted with caution, in view of these inherent difficulties in their generalizability. It is also possible that our participants provided socially acceptable answers, particularly regarding the adherence to consultations among CAG members, and that their retention to care is actually somewhat less ideal than at first appearance. Regarding their information on barriers to care, however, socially acceptable answers were much less obvious, and, in addition, saturation of information was reached rather early in the series of debates, while subsequent discussions repeatedly confirmed the earlier findings. Despite these drawbacks, we therefore feel that our findings, both quantitative and qualitative, provide a reasonably accurate picture of the utilization of and barriers to utilization of HIV and MCH care amongst the CAG members and their family aggregates in Changara and possibly suggest similar tendencies among comparable populations elsewhere. 

Community participation as a bottom-up strategy, binding people on the basis of their common needs, engaging people in their lifelong care, and increasing awareness of the needs of their fellow community members has a potential in Sub-Saharan contexts, where social capital is a means to survive in its harsh economic environments. People support each other knowing that roles can change over time and that the supporting may become the supported [21, 22]. These traditional ways to overcome barriers can become the foundations on which health interventions can be built. It is important to engage the real decision makers (both male and female) within the communities, such as traditional chiefs, church leaders, and other people with a credibility which is widely recognized. Some of the participating CAG members are well known in their community, as they use their service utilization experiences to guide others who suffer disease or face other health needs. We therefore stress the importance of linking HIV care, including the very successful and innovative models such as the CAG, with broader SRH, including MCH, activities, and services, leading to the promotion of comprehensive family-centred care.

## Figures and Tables

**Table 1:** Demographic characteristics of the participants.

	Formal and social CAG	Non-CAG
Number of family aggregate members reached	420 (26%)	1 204 (76%)
Range of members per family aggregate	1 to 5	0 to 13
Sex distribution	302 females (72%) 118 males (28%)	570 females (47%) 634 males (53%)
Number of women of reproductive age (15–49 years)	255 (61%)	160 (13%)
Number of children under five years of age	4 (1%)	204 (17%)

**Table 2:** Utilization of HIV services.

	Formal and social CAG members (*N* = 420)	Non-CAG members (1,204 persons; 1,199 with known age)				
		Men aged 15 years or more (*N* = 252)	Women aged 15 years or more (*N* = 200)	Children aged 6–14 years (*N* = 544)	Children aged 0–5 years (*N* = 203)	HIV-exposed infants aged 0–18 months (*N* = 34)
Counselling and testing for HIV	420/420 (100%)	77/243 (32%)	81/195 (41%)	158/357 (44%)	89/179 (50%)	16/24 (67%)
Tested HIV positive	420/420 (100%)	14/75 (19%)	13/79 (16%)	8/149 (5%)	4/84 (5%)	0/14 (0%)
HIV treatment and care:						—
ART care	358/420 (85%)	10/14 (71%)	3/13 (23%)	4/8 (50%)	1/4 (25%)	
OI care	62/420 (15%)	1/14 (7%)	6/13 (46%)	4/8 (50%)	2/4 (50%)	
None	0/420 (0%)	3/14 (21%)	4/13 (31%)	0/8 (0%)	1/4 (25%)^2^	
Retention in HIV care (consultation in last 6 months)	420/420 (100%) within previous 6 months	8/8 (100%) within previous 6 months	8/8 (100%) within previous 6 months	8/8 (100%) within previous 6 months	3/3 (100%) within previous 6 months	27/34 (79%) registered in follow-up programme

OI: opportunistic infections; ART: antiretroviral therapy.

Denominators vary as information was not available for all variables and all participants.

**Table 3:** Healthcare for women of reproductive age.

	Women of reproductive age (formal and social CAG members; 255 women)	Women of reproductive age (non-CAG members; 160 women)
Pregnancy	18/247 (7%) report a current pregnancy 11/18 (61%) started antenatal care 18/18 (100%) are HIV-positive pregnant women 16/18 (89%) receive PMTCT	7/153 (5%) report a current pregnancy 6/7 (86%) started antenatal care 2/7 (29%) are HIV-positive pregnant women 0/2 (0%) receive PMTCT

Childbirth	227/255 (89%) report at least one childbirth 176/227 (78%) report skilled attendance	88/160 (55%) report at least one childbirth 73/88 (83%) report skilled attendance

Contraceptive use	37/226 (16%) report current use of a modern method	24/126 (19%) report current use of a modern method

Tetanus immunization	45/109 (41%) with up-to-date immunization	14/37 (38%) with up-to-date immunization

Denominators vary as information was not available for all variables and all participants.

**Table 4:** Healthcare for children under five years of age (including 4 CAG and 204 non-CAG members).

	Children aged 0–11 months (41 children)	Children aged 12–59 months (167 children)
Fully immunized for age	21/29 (72%)	81/85 (95%)
Growth monitoring	23/29 (79%) within the previous month	43/85 (52%) within the previous 3 months
Low weight-for-age in the last consultation	4/29 (15%); of whom 3 (75%) were referred for further evaluation and care	43/87 (49%); of whom 10 (23%) were referred for further evaluation and care
Exclusive breastfeeding (age <6 months)	14/20 (70%)	—
Vitamin A supplementation (age 6–59 months)		61/98 (62%) up-to-date
Deworming (age 12–59 months)	—	52/85 (61%) up-to-date

Denominators vary as information was not available for all variables and all participants.
